# Analysis of the Impact of Dexmedetomidine Use in Very Preterm Infants on Long-Term Neurodevelopmental Outcomes

**DOI:** 10.7759/cureus.86005

**Published:** 2025-06-14

**Authors:** Erin Cicalese, Aashish Shah, Ferras Bashqoy, Kristyn Pierce, Heather Howell, Purnahamsi Desai

**Affiliations:** 1 Neonatology, New York University (NYU) Grossman School of Medicine, New York, USA; 2 Pediatrics, New York University (NYU) Grossman School of Medicine, New York, USA

**Keywords:** neonatal intensive care unit (nicu), neurological outcomes, opioids, opioid side effects, precedex, premature, premature infants

## Abstract

Background: Prolonged sedation in premature infants often involves opioids and benzodiazepines, which can cause adverse effects and worse neurodevelopmental outcomes. Dexmedetomidine has emerged as a safer alternative with fewer side effects, but its long-term neurodevelopmental impact on very preterm infants remains unclear. Further research is needed to understand its effects on this vulnerable population.

Objectives: The primary objective is to compare the neurodevelopmental outcomes of premature infants who received and did not receive dexmedetomidine infusions while intubated. The secondary objective is to compare the rate of unplanned extubations in these groups.

Methods: This was a retrospective cohort study deemed Institutional Review Board (IRB)-exempted by the New York University (NYU) IRB. The study population (n = 15) with a matched control cohort (n = 15) includes infants born under 32 weeks gestation or weighing less than 1,500 g who were intubated during their hospitalization and followed up at our high-risk follow-up program. Patients excluded from the study include those who did not survive to discharge, those lost to follow-up, or those with major congenital anomalies. The patient chart was reviewed for data on maternal characteristics and details from the infant’s neonatal intensive care unit (NICU) admission. Data from follow-up visits at six months to two years of life included the Bayley Scales of Infant and Toddler Development, Fourth Edition (BSID-IV) scores. Data were analyzed using Mann-Whitney U testing and Fisher’s exact testing.

Results: There was no statistically significant difference (p = 0.373) in BSID-IV scores between the two groups. The overall number of unplanned extubations was not different between the two groups. When assessing unplanned extubations per intubation day, there was a trend toward fewer unplanned extubations in the dexmedetomidine group.

Conclusions: This study suggests that dexmedetomidine may be a safe and effective alternative to traditional sedatives for extremely premature infants, with no observed adverse effects on long-term neurodevelopmental outcomes and potential benefits in reducing extubation-related complications. However, larger, multi-center prospective studies are needed to confirm these findings and inform clinical practice.

## Introduction

Premature infants often require sedation to reduce complications and enhance comfort during uncomfortable procedures [1]. Nevertheless, existing literature suggests that exposure to sedative drugs may have adverse effects on the developing brain [2,3]. Clinicians are faced with the challenge of balancing the benefits of short-term comfort against potential long-term developmental risks [4]. Historically, morphine and fentanyl have been the primary sedatives in neonatal intensive care units (NICUs) across the United States [5]. However, recent concerns regarding the negative impacts of opioids on the central nervous system, particularly in extremely premature populations, have prompted the exploration of alternative sedatives [6,7].

Dexmedetomidine, a highly selective α-2 adrenergic agonist, is known for its sedative properties that work by reducing sympathetic outflow [8]. Research has established dexmedetomidine's potential benefits in neonates, including a decreased reliance on opioids and benzodiazepines, agents associated with detrimental effects on brain development [9,10]. Additionally, animal studies indicate that dexmedetomidine possesses neuroprotective properties, facilitates shorter mechanical ventilation durations, and promotes earlier enteral feedings, which could help with better growth and potentially reduce necrotizing enterocolitis [11]. However, long-term neurodevelopmental outcomes in premature infants sedated with dexmedetomidine remain unexplored in current literature.

Dexmedetomidine has been adopted as the primary sedative for premature infants since 2022 in our level IV NICU. Of these, 15 infants have transitioned from the NICU and participated in neurodevelopmental follow-up evaluations at the Neonatal Comprehensive Care Program (NCCP) clinic at New York University (NYU), where standardized developmental screenings were conducted. The data accumulated from these follow-up visits, ranging from age six months to two years, provide an opportunity to evaluate the long-term neurodevelopmental outcomes of extremely premature infants who received dexmedetomidine as first-line sedation. We hypothesize that there will be no significant long-term neurodevelopmental differences, as measured by standardized testing at follow-up clinic visits, between extremely premature infants who received dexmedetomidine and those who did not receive dexmedetomidine. Furthermore, we expect there to be no difference in unplanned extubations for those who received dexmedetomidine as first-line sedation.

## Materials and methods

This study was designed as a retrospective non-inferiority cohort study that was deemed exempt from full review by the NYU Institutional Review Board (IRB). The study population consisted of infants born at less than 32 weeks gestation or weighing less than 1,500 g, all of whom were intubated during their stay in the NICU. These infants were subsequently followed up at our high-risk follow-up program between six months and two years of life. Eligible participants were identified through a review of NICU admission records from January 1, 2020, to January 1, 2024.

**Figure 1:**
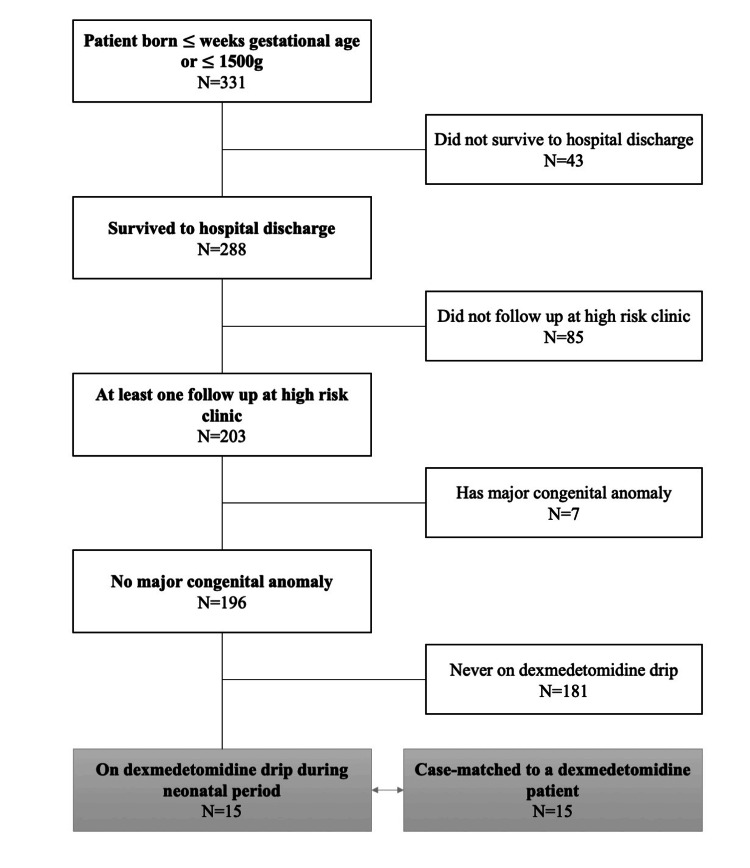
Patient flow sheet

The inclusion criteria for this study were infants who were admitted to our NICU and met the gestational age or weight thresholds specified above. Infants were excluded if they did not survive to discharge, were lost to follow-up after discharge, or were diagnosed with major congenital anomalies that could potentially confound neurodevelopmental outcomes. The study cohort consisted of patients who received dexmedetomidine as primary sedation medication. The matched control cohort, based on gestational age, weight, and intubation status, included patients who had no sedation or other primary sedation, mainly opioid or benzodiazepine (Figure 1).

Data were extracted from patient charts including details from the infant’s hospitalization. The information collected encompassed the method of sedation utilized, duration of mechanical ventilation, and other significant clinical variables relevant to the NICU course. During the follow-up visits at NCCP, developmental outcomes were assessed using the Bayley Scales of Infant and Toddler Development, Fourth Edition (BSID-IV), which provides standardized scores in three domains: cognitive, language, and motor skills.

Continuous variables were assessed using the Mann-Whitney U test to compare differences in BSID-IV scores between the cohorts. Categorical variables were analyzed using Fisher's exact test. A significance level of p < 0.05 was set for all statistical tests.

## Results

Baseline demographic data of the control group (n = 15) and dexmedetomidine group (n = 15) are shown in Table 1, with no statistically significant differences between the two groups.

**Table 1:** Basic demographics

Birth characteristics	Control group (n = 15)	Dexmedetomidine group (n = 15)	p-value
Gestational age (weeks), median (IQR)	25.4 (24.3, 26.4)	25.2 (24.6, 27.2)	0.175
Birth weight (g), median (IQR)	752 (590, 1,000)	760 (592, 1,000)	>0.999
Birth length (cm), median (IQR)	31 (29, 35.5)	32.0 (30, 33.5)	0.663
Birth head circumference (cm), median (IQR)	31 (21, 35)	23 (21, 30)	0.254
Male, n (%)	4 (26.7%)	7 (46.7%)	0.45

Additionally, baseline clinical data (Table 2) show the two groups are similar in medical complexity and acuity.

**Table 2:** Baseline clinical data *Score for Neonatal Acute Physiology with Perinatal Extension-II (SNAPPE-II) APGAR: appearance, pulse, grimace, activity, and respiration

Birth data	Control group (n = 15)	Dexmedetomidine group (n = 15)	p-value
SNAPPE-II* score, mean (IQR)	14 (12, 30)	16 (14, 36)	0.237
1 minute APGAR, median (IQR)	5 (4, 7)	5 (3, 7)	0.885
5 minute APGAR, median (IQR)	8 (7, 8)	7 (7, 9)	0.74
# of total days intubated, median (IQR)	17 (3, 45)	56 (24, 73)	0.062
Intubated in delivery room (n)	6 (40%)	5 (33.3%)	>0.999
# of intubations (n), median (IQR)	1 (1, 3)	2 (1, 3)	0.31
Head ultrasound with grade 3 or 4 (n)	1 (6.7%)	1 (6.7%)	>0.999

The median duration of dexmedetomidine use was 30 days (IQR 12.5-54.5) with a median initial dose of 0.3 mcg/kg/hr (IQR 0.25-0.3) and the highest median dose of 0.6 mcg/kg/hr (IQR 0.3-1). The primary outcome of developmental assessment measured using BSID-IV showed no statistically significant differences between the control and the dexmedetomidine groups (Table 3).

**Table 3:** Primary outcome: neurodevelopmental data

Bayley Scales of Infant and Toddler Development (BSID) III and IV scores	Control group (n = 15)	Dexmedetomidine group (n = 15)	p-value
Cognitive composite, mean	100 (100, 105)	100 (90, 100)	0.373
Language composite, mean	95 (80, 106)	92 (86, 100)	0.868
Receptive language SS, mean	9 (6, 11)	9 (7, 10)	0.934
Expressive language SS, mean	9 (6, 11)	9 (8, 11)	0.967
Motor composite, mean	97 (88, 101)	90 (84, 102)	0.633
Fine motor SS, mean	9 (9, 11)	9 (8, 11)	0.633
Gross motor SS, mean	9 (7, 11)	9 (7, 11)	0.917
BSID-III and IV scores < 85	Control group (n = 15)	Dexmedetomidine group (n = 15)	p-value
Language composite (n)	4 (26.7%)	3 (20%)	>0.999
Motor composite (n)	3 (20%)	5 (33.3%)	0.682

Analyzing the secondary outcome of unplanned extubations, the number of unplanned extubations did not differ significantly between the two groups (Table 4).

**Table 4:** Secondary outcome: unplanned extubations

Intubation data	Control group (n = 15)	Dexmedetomidine group (n = 15)	p-value
# of patients with unplanned extubations, n (%)	2 (13.3%)	3 (20%)	>0.999
# of unplanned extubations per intubation day	5/455 = 0.01	4/877 = 0.005	0.29

However, when analyzing the data per intubation day, a trend emerged indicating potentially fewer unplanned extubations in the dexmedetomidine group. Finally, the amount of opioid or benzodiazepine exposure over the first 30 days of life was not significantly different between the two groups (Table 5).

**Table 5:** Opioid and benzodiazepine exposure

Intubation data	Control group (n = 15)	Dexmedetomidine group (n = 15)	p-value
# of patients with unplanned extubations, n (%)	2 (13.3%)	3 (20%)	>0.999
# of unplanned extubations per intubation day	5/455 = 0.01	4/877 = 0.005	0.29

## Discussion

Dexmedetomidine is a selective α-2 agonist that induces sedation by reducing sympathetic outflow and provides analgesia by inhibiting substance P; it offers advantages over benzodiazepines and opioids by providing sedation and analgesia with minimal respiratory depression and greater hemodynamic stability. Because of that, it has become a popular first-line sedation choice in NICUs, but there is still a lack of long-term neurodevelopmental data, as exists for opioids and benzodiazepines [12-14]. Our data demonstrate that there is no significant difference in neurodevelopmental outcomes between very preterm infants who received dexmedetomidine infusions and those who did not. This finding is important as it indicates that the use of dexmedetomidine for sedation does not adversely affect the neurodevelopment of this vulnerable population. Additionally, our analysis showed no difference in the rate of unplanned extubations between the two groups. This suggests that dexmedetomidine may be a safe sedation option that does not increase the risk of respiratory complications commonly associated with mechanical ventilation.

These results contribute to the growing body of evidence supporting dexmedetomidine use for sedation in very preterm infants in the NICU. By demonstrating both safety in terms of neurodevelopment and respiratory stability, these data reinforce the potential of dexmedetomidine as an effective sedation strategy in clinical practice. As the field continues to explore optimal sedation protocols, these findings highlight the necessity for further research into long-term outcomes and the effects of different dosing regimens of dexmedetomidine. Such studies could provide deeper insights into its safety and efficacy, ultimately enhancing care practices for very preterm infants in the NICU.

An essential aspect of neonatal sedation involves balancing the prevention of unplanned extubations with the need to avoid oversedation [15]. Prior studies have demonstrated that neonates who undergo unplanned extubations face higher risks of requiring tracheostomies, experience prolonged hospitalizations, and develop anatomical complications such as subglottic stenosis [16]. Additionally, these infants are more susceptible to bronchopulmonary dysplasia and may suffer from poorer neurodevelopmental outcomes [17]. Encouragingly, transitioning from traditional sedatives like opioids and benzodiazepines to dexmedetomidine has not resulted in an increase in unplanned extubation events. Preliminary data suggest a trend toward fewer unplanned extubations per total intubated days with this transition, although further studies with larger sample sizes are warranted to achieve statistical significance. It is both reassuring and encouraging that the transition of sedation from opioids and benzodiazepines to dexmedetomidine shows no different events of unplanned extubations. Data show there is a trend toward fewer unplanned extubations per total intubated days, though a higher sample size would be needed for statistical significance.

This study has several limitations that must be acknowledged. Firstly, it was a retrospective, single-center study, which inherently limits the generalizability of our findings. Secondly, a significant number of patients were lost to follow-up, which emerged as the largest reason for exclusion from the analysis. This loss could introduce bias and affect the reliability of our results. The extent to which these excluded patients differed from those included in the study is unknown and may influence the observed outcomes. Finally, the relatively small sample size of our study may have reduced the statistical power to detect subtle differences or trends, particularly in secondary outcomes. While we noted a trend toward fewer unplanned extubations in the dexmedetomidine group, the limited number of participants means that such findings should be interpreted with caution. Future research should aim to include larger cohorts to enhance statistical robustness and provide more definitive conclusions about the use of dexmedetomidine in this population.

## Conclusions

The study findings suggest that dexmedetomidine serves as a promising alternative to traditional opioid sedatives in the care of extremely premature infants within the NICU. There has been a lack of studies looking at the long-term neurodevelopmental outcomes of premature infants who received dexmedetomidine as a first-line agent for sedation. This study provides reassuring evidence that dexmedetomidine can be utilized in this vulnerable population without the developmental risks associated with opioids. Furthermore, while the rate of unplanned extubations did not significantly differ between those receiving dexmedetomidine and those who did not, a favorable trend suggests potential benefits in reducing extubation-related complications. This aspect is particularly important given the respiratory challenges faced by premature infants and the potential for dexmedetomidine to support stability during their NICU stay.

However, despite these positive indications, it is essential to approach these findings with consideration of the study's limitations. The retrospective, single-center nature of the research limits broad applicability, and the relatively small sample size may mask subtle neurodevelopmental differences and trends in secondary outcomes. The loss of patients to follow-up poses additional challenges in ensuring the robustness of our results. Future research should aim to address these limitations by incorporating larger, multi-center cohorts and prospective methodologies, thus enhancing the statistical power and generalizability of findings. In doing so, the medical community can attain a more comprehensive understanding of the long-term impacts and potential benefits of dexmedetomidine in neonatal care, ultimately refining sedation protocols for the betterment of outcomes in premature infants.
